# Application of Hyperspectral Imaging and Chemometric Calibrations for Variety Discrimination of Maize Seeds

**DOI:** 10.3390/s121217234

**Published:** 2012-12-12

**Authors:** Xiaolei Zhang, Fei Liu, Yong He, Xiaoli Li

**Affiliations:** 1 College of Biosystems Engineering and Food Science, Zhejiang University, 866 Yuhangtang Road, Hangzhou 310058, China; E-Mails: xiaoleizhang@zju.edu.cn (X.Z.); fliu@zju.edu.cn (F.L.); xiaolili@zju.edu.cn (X.L.); 2 Cyrus Tang Center for Sensor Materials and Applications, Zhejiang University, 866 Yuhangtang Road, Hangzhou 310058, China; 3 Key Laboratory of Equipment and Informatization in Environment Controlled Agriculture, Ministry of Agriculture, 866 Yuhangtang Road, Hangzhou 310058, China

**Keywords:** maize seed, variety identification, hyperspectral imaging, principal component analysis, kernel principal component analysis, gray-level co-occurrence, least squares-support vector machine, back propagation neural network

## Abstract

Hyperspectral imaging in the visible and near infrared (VIS-NIR) region was used to develop a novel method for discriminating different varieties of commodity maize seeds. Firstly, hyperspectral images of 330 samples of six varieties of maize seeds were acquired using a hyperspectral imaging system in the 380–1,030 nm wavelength range. Secondly, principal component analysis (PCA) and kernel principal component analysis (KPCA) were used to explore the internal structure of the spectral data. Thirdly, three optimal wavelengths (523, 579 and 863 nm) were selected by implementing PCA directly on each image. Then four textural variables including contrast, homogeneity, energy and correlation were extracted from gray level co-occurrence matrix (GLCM) of each monochromatic image based on the optimal wavelengths. Finally, several models for maize seeds identification were established by least squares-support vector machine (LS-SVM) and back propagation neural network (BPNN) using four different combinations of principal components (PCs), kernel principal components (KPCs) and textural features as input variables, respectively. The recognition accuracy achieved in the PCA-GLCM-LS-SVM model (98.89%) was the most satisfactory one. We conclude that hyperspectral imaging combined with texture analysis can be implemented for fast classification of different varieties of maize seeds.

## 1. Introduction

Effective variety discrimination of maize seeds is increasingly vital for the growing food industry owing to the appearance on the market of more and more new maize varieties like Sweet maize, Waxy maize, Popcorn, Dent maize and Amylomaize, during these years. Different varieties of maize seeds have different characteristics and qualities. Types of maize are commonly classified depending on their quality parameters, such as oil content, sweetness, and degree of waxiness. How to target and recommend an appropriate maize variety which meets the varietal purity standards for target markets is a serious problem faced by variety breeders, farmers, bulk handlers, marketers, and others. However, the traditional and prevailing methods for seed cultivar identification, like grain morphology, fluorescent scanning, protein electrophoresis and DNA molecular markers are time consuming, expensive, complex to use and subject to human error and inconsistency. To overcome these shortcomings, an approach for quickly and reliably identifying maize seed varieties would be highly desirable and beneficial from both technical and economical points of view. Thus, is this work automatic variety identification based on hyperspectral imaging technique was investigated.

Hyperspectral imaging is an emerging platform technology that integrates spatial information, as regular imaging systems, and spectral information for each pixel in the image. Compared to conventional RGB imaging, NIR spectroscopy and multispectral imaging, hyperspectral imaging has many advantages, like containing spatial, spectral and multi-constituent information and sensitivity to minor components [1]. The combined nature of imaging and spectroscopy in hyperspectral imaging enables this system to provide images in a three-dimensional (3-D) form called “hypercube” which can be analysed to ascertain minor and/or subtle physical and chemical characteristics of a sample as well as their spatial distributions. This technique, originally developed for remote sensing applications [2], has since found application for non-destructive food analysis [3–6].

Regarding the classification of agricultural products, the technique has been successfully applied in detection on apples [7–9] and cucumbers [10–12]. Moreover, hyperspectral imaging found its way for potential applications in evaluation of cereal quality such as wheat classes identification [13], insect fragments assessment in flour milled from infested wheat [14], maize kernel hardness classification [15] and early detection of toxigenic fungi in maize [16].

Although many studies have been focused on wheat and rice variety identification and quality inspection, no research endeavours using hyperspectral imaging have been reported for maize seeds. Therefore, it is our interest to implement this technology to aid visual inspection and replace human judgement in the discrimination of different seeds. The aim of this study was to investigate the feasibility of using hyperspectral imaging in the 380–1,030 nm visible and near infrared spectral region for the variety discrimination of maize seeds. The specific objectives were to: (1) extract spectral features from the average reflectance spectrum of hyperspectral images using principal component analysis (PCA) and kernel principal component analysis (KPCA); (2) extract texture features from hyperspectral images using PCA and Gray-level co-occurrence matrix (GLCM); (3) develop several classification models using least squares-support vector machine (LS-SVM) and back propagation neural network (BPNN) based on different combinations of spectral features and texture features, respectively, and (4) obtain an optimal calibration model after comparing the performance of different algorithms.

## 2. Experimental Section

### 2.1. Sample Preparation

A total of 330 samples of six maize seed varieties were collected from the Seed Company of Zhejiang Province in China, including Heinuo (I), Huyunuo (II), Sukehuanuo (III), Jinyin (IV), Meiyu (V) and Suyu (VI). These six varieties of maize seed were all produced in Zhejiang Province in 2010. There were different cultivar registrated codes among these different brands according to Maize GB1353-2009, State Standard of the People’s Republic of China. This classification method is mainly based on the testa colour. Maize seeds were evenly distributed in glass dishes of the same size (∅120 mm × 10 mm), and the surface of samples was smoothed. Each dish was then imaged individually in the hyperspectral imaging system as explained below.

### 2.2. Hyperspectral Imaging System

**Figure 1.**
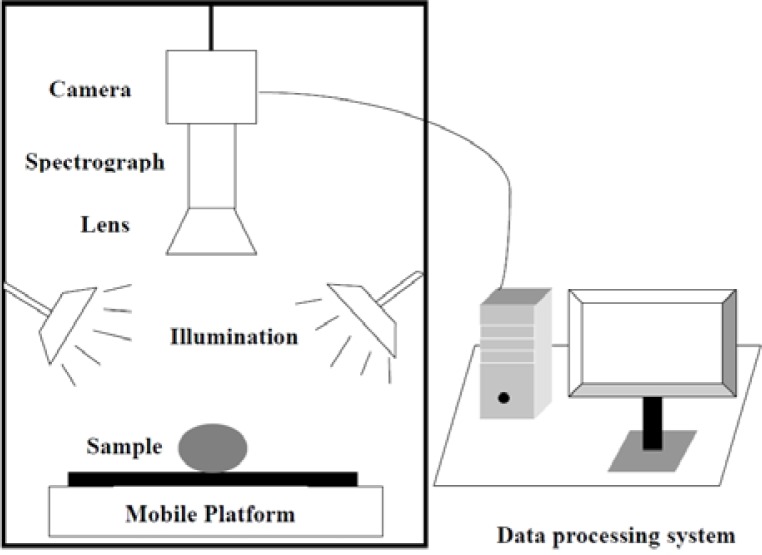
Schematic diagram of hyperspectral imaging system.

A laboratory visible and near infrared (VIS-NIR) hyperspectral imaging system was assembled to acquire hyperspectral images for maize seeds. As shown in Figure 1, the hyperspectral imaging system consists of a imaging spectrograph (ImSpectorV10, Spectral Imaging Ltd., Oulu, Finland), a high performance CCD camera (C8484-05; Hamamatsu, Hamamatsu City, Japan), an illumination unit containing two 150 W quartz tungsten halogen lamps (2900ER; Illumination Technologies, Inc., New York, NY, USA), a mobile platform used for samples removing and a computer running the Spectral Cube data acquisition software (V10E and N17EIsuzu Optics Corp., Taiwan, China) which controls the motor speed, exposure time, binning mode, wavelength range and image acquisition. The ground track of mobile platform was 300 mm. The camera spectral range was from 380 nm to 1030 nm divided in 512 bands. The camera has 672 × 512 (spatial × spectral) pixels with a spectral resolution of 2.8 nm.

### 2.3. Image Acquisition

Each glass dish filled with seeds was placed on the mobile platform and then moved at a speed of 4.5 mm/s to be scanned using 0.06 s exposure time to build a hyperspectral image with dimensions (*x*, *y*, *λ*), where *x* and *y* are the spatial dimensions (number of rows and columns in pixels) and *λ* is the number of wavebands. Therefore, the images were acquired with 672 pixels in *x*-direction, *n*-pixels in *y*-direction (based on the length of the sample) and 512 wavelengths in *λ*-direction with 1.23 nm between contiguous bands. 100 × 100 pixels were randomly selected from each image as a region of interest (ROI) and also treated as one sample. About 10 ROIs were produced in one image and totally 330 ROIs, *i.e.*, 330 samples were used to extract the spectral features and texture features

### 2.4. Image Correction

For calculating the reflectance spectrum, the spectral raw images (*I_0_*) of the samples were corrected using two reference standards: a “white” one (*W*) to set-up the maximum reflectance conditions, which was obtained for a Teflon white surface under the same condition of the raw image; and a “black” one (*B*) to define the no reflectance condition (zero), which was acquired by turning off the light source and completely covering the lens with its opaque cap. The calibrated image (*I*) was then calculated using the following equation:

$$I=\frac{I_{0}-B}{W-B}$$

**Figure 2.**
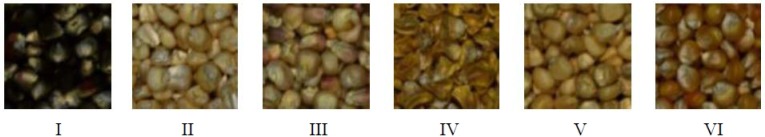
Images acquired from six varieties of maize seeds.

Figure 2 shows the corrected ROI images of six varieties of maize seeds after this correction process. It could be seen that the colour of I was almost black. Type II and V were similar and pale yellow. IV and VI were approximately orange. Sample III showed a little pink in yellow. Besides, variety IV showed obviously different shape. These corrected images will be the basis for the subsequent image analysis to extract the spectral properties and textural features variables. All the processing and analyzing of the acquired hyperspectral data were carried out using the Environment for Visualizing Images (ENVI 4.6) software (ITT visual information solutions, Boulder, CO, USA).

### 2.5. Data Analysis

#### 2.5.1. Principal Component Analysis

PCA is a multivariate statistical tool developed primarily to obtain a parsimonious representation of multivariate data. Orthogonal transformation by PCA results in fewer independent variables but maximum representation of original variables [11]. In this study, reflectance values of all pixels identified by each ROI were averaged to produce one mean value for each band, and the whole 512 mean values of 512 bands represented the average reflectance spectrum of each sample. The same routine was repeated for all ROI images of all samples. Then the spectral data extracted from 330 samples of different varieties were firstly analyzed using PCA. The full cross validation method was used for PCA. PCA applied on average spectra was implemented using “The Unscrambler V9.7 software” (CAMO PROCESS, AS, Oslo, Norway).

PCA was also directly employed on the selected ROI images to create the PC images using ENVI software. Each PC image is a linear sum of the original images at individual wavelengths multiplied by corresponding (spectral) weighting coefficients [17]. Although multivariate data analysis can sometimes be applied directly to data of continuous spectra, its calibration process is often time-consuming [18]. Loadings resulting from PCA (weighting coefficients) can be used to identify important variables that are responsible for the specific features appearing in the corresponding scores. To remove redundant information for realizing hyperspectral imaging in potential on-line inspections, some optimal wavelengths were selected. According to previous research, optimal wavelengths may be equally or more efficient than full wavelengths [19,20]. The reduced number of wavelengths is enough to characterize most of the classification tasks [21]. Therefore, several wavelengths with high (local maxima) and low (local minimum) weighing coefficients from the PC loadings were selected as the dominant wavelengths [22]. Additionally the monochromatic images of these optimal wavelengths were then selected as the optimum images to represent the most significant variance and loading weights for classifying six cultivars of maize seeds.

#### 2.5.2. Kernel Principal Component Analysis

In order to compare with PCA, another reduction dimension approach, kernel principal component analysis (KPCA), was implemented to extract the spectral features. KPCA successfully extends PCA to nonlinear cases by performing PCA in a higher or even infinite dimensional feature space which is nonlinearly transformed from input space and implicitly defined by a kernel function [23]. The idea of KPCA is to firstly map the original data *X* = [*x*_1_, …,*x*_n_], *n* = 1, …,*N*, into a high-dimensional feature space *F* using a nonlinear mapping *ϕ: R*^P^→*F*, and then the linear PCA is executed in *F* based on the mapped data *φ*(*x*_n_) [24]. In this study, the powerful kernel function of gaussian radial basis (RBF) is adopted for KPCA. The first few optimal kernel principal components (KPCs) and principal components (PCs) would be selected as the inputs variables to develop classification models, respectively. KPCA was realized by MATLAB 7.8.0.347 (R2009a) software (The Mathworks, Inc., Natick, MA, USA).

#### 2.5.3. Gray-Level Co-Occurrence Matrix

GLCM analysis was executed to extract second-order statistical textural features variables from the PC images using each of the selected dominant wavelengths. GLCM is a statistical technique for texture analysis. Probably, the most frequently cited method for texture analysis is based on extracting various textural features from a GLCM. A general procedure for extracting textural features of image in the spatial domain was presented by Haralick *et al*. [25]. A co-occurrence matrix is a square matrix with elements corresponding to the relative frequency of occurrence of pairs of grey level of pixels separated by a certain distance in a given direction (0°, 45°, 90°, or 135°) [26]. In this study, the textural features were calculated from the GLCM when the direction equals to 0° and the distance equals to 1, respectively. The following four GLCM parameters were calculated using a program developed by MATLAB 7.8 to express texture: contrast, homogeneity, energy and correlation. Then these textural variables from the optimal PC images and the PCs or KPCs selected from average spectrum data mentioned before were implemented together as the inputs of LS-SVM and BPNN to build classification models, respectively.

#### 2.5.4. Least Squares-Support Vector Machine

LS-SVM is a state-of-the-art statistical algorithm capable of learning in high-dimensional characteristic space with fewer training variables or samples [27–29]. It uses a linear set of equations instead of a quadratic programming (QP) problem to obtain the support vectors (SVs). Successful examples of LS-SVM applications for quantification and classification have been reported [30–33]. In this study, total 330 samples were randomly split into two groups, 240 samples (40 of each variety) of which were selected for the calibration set, and the remaining 90 samples (15 of each variety) were applied as the prediction set. As giving a good performance under general smoothness assumptions on handling the nonlinear relationships between the spectra and target attributes, RBF kernel was used in this study [34]. The free LS-SVM toolbox (LS-SVM v 1.5, Suykens, Leuven, Belgium) was applied with MATLAB to develop the calibration models.

#### 2.5.5. Back Propagation Neural Network

In order to compare the performance of LS-SVM models, BPNN was applied in this study. BPNN is a type of nonlinear neural network used to solve several types of classification and regression problems. The eigenvectors obtained from compressing the raw spectra were processed by the neural network and the network output expresses the resemblance that an object corresponds with a training pattern [35].The theory of BPNN has been described extensively [36,37]. All calculations of BPNN were carried out based on the Neural Networks toolbox of MATLAB.

## 3. Results and Discussion

### 3.1. Spectral Analysis

#### 3.1.1. Reflectance Spectra of Maize Seeds

**Figure 3.**
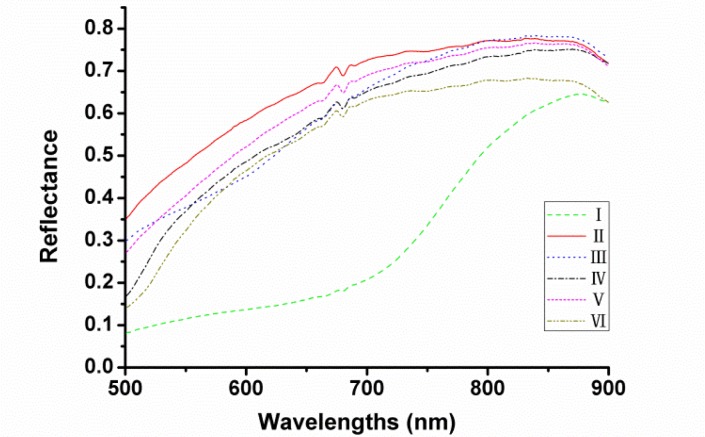
Vis/NIR reflectance of six different maize seeds extracted from the ROI pixels of hyperspectral images.

The actual optical sensitivity of this system ranges from 380 to 1,030 nm but only the range of 500–900 nm was used to avoid low signal-to-noise ratio. The average reflectance spectra of each variety of seeds in the spectral range of 500–900 nm are shown in Figure 3 respectively. It can be seen that the trends of the spectral curves were quite similar except the one of cultivar I (Heinuo) since this variety looked almost black while others appeared approximately yellow. However, it could not distinguish all cultivars of maize seeds by colour variance. Therefore, further treatments would be needed and then the latent features of the spectra could be applied for the variety discrimination of maize seeds.

#### 3.1.2. PCA Using Spectral Data

**Figure 4.**
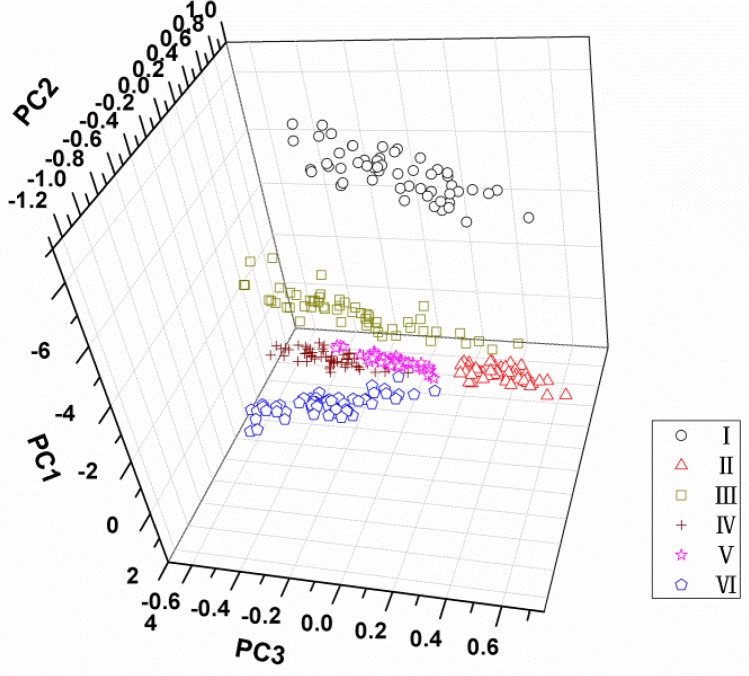
Score cluster plot with PC1× PC2 × PC3 of each maize variety.

PCA was applied on all spectral data (500–900 nm) acquired from all samples to reduce the high dimensionality and to check qualitative discrimination in the spectra among the maize seeds. The explained variance rate for the first three principal components was 95%, 3% and 1% of the total variance, respectively. It indicated that the cumulative reliabilities of the first three PCs could explain 99% of the total information, so they could be used to represent the 315 variables for classification of maize seeds. The interpretation of the results of PCA is usually carried out by visualization of its PC scores. Figure 4 shows the scores plot of PC1 × PC2 × PC3 of total samples. It can be found that different varieties distributed separately in the three-dimension area, and variety I seems far away from the other five cultivars owing to its distinct colour. However, although the sample points of varieties from II to VI were clustered, respectively, their borders are not clear and some sample points near the borders are mixed. For this, it is hard to distinguish all kinds of samples in the three-dimension area of PC scores plot. Therefore, more classifiers were needed based on the PCA process.

#### 3.1.3. KPCA Using Spectral Data

Similarly, KPCA was used in the spectral region between 500 nm and 900 nm. The top three KPCs were extracted and they could explain 99.63% variance of all features, which corresponding to the accumulative variance of 99% from the first three PCs by PCA. It sketched that the first three KPCs could also express the total spectral information and the KPCA feature extraction method is a little more superior to the traditional PCA method.

### 3.2. Textural Analysis

#### 3.2.1. Selection of Effective Wavelengths

As stated above, PCA directly implementing on each ROI image using ENVI was used for identification of optimal wavelengths. The PC loadings can be used to identify sensitive wavelengths that are highly correlated with each PC’s.

**Figure 5.**
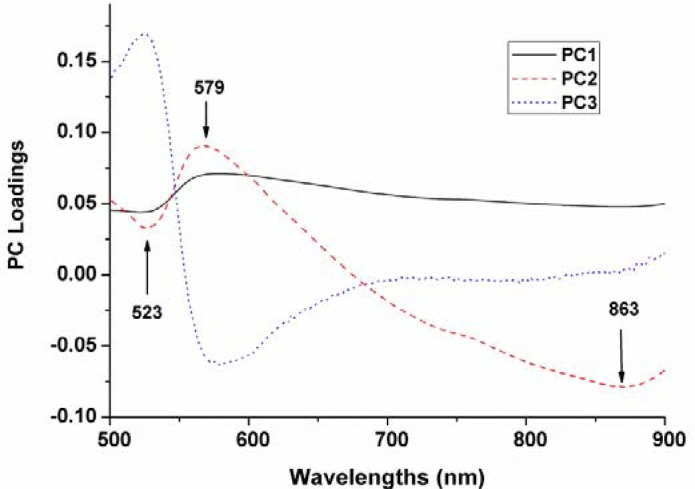
Loading weights of the first three PCs from PCA on ROI images for selecting optimal wavelengths.

The top three PCs were used for x-loading weights to select wavelength in the entire spectral range. The wavelengths corresponding to peaks (maxima) and valleys (minima) at these particular principal components were selected as optimum wavelengths (Figure 5). Therefore, three wavelengths (523, 579 and 863 nm) were then selected as the effective wavelengths which can later be used to discriminate the different varieties of maize seeds. Such reduced number of wavelengths would help in decreasing the time required to acquire and process each spectral images.

#### 3.2.2. Textural Feature Extraction from GLCM

**Figure 6.**
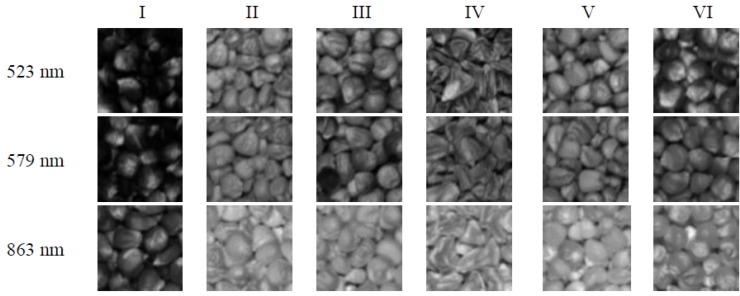
Monochrome images obtained using three selected optimal wavelengths.

The wavelengths selected before may represent the differences of colour and different content of ingredients in maize seeds. Thus, the monochromatic images of the effective wavelengths were then selected as the optimal images to represent the most significant variance and loading weights within the whole region. Four textural features including contrast, homogeneity, energy and correlation were calculated from GLCM of each monochromatic image. Additionally, there were three monochromatic images for each sample corresponding to optimal wavelengths 540 nm, 670 nm, and 800 nm, so 12 textural features were then generated for one sample through GLCM feature extraction. Figure 6 is the monochromatic images of six varieties at three sensitive wavelengths. The development of LS-SVM models and BPNN models were based on the four combinations of these 330 × 12 textural variables, PCs and KPCAs resulting from the average spectra. Specifically, the different input combinations were PCs, PCs combined with textural features, KPCs and KPCs combined with textural features, respectively.

### 3.3. Maize Seeds Classification by LS-SVM and BPNN Models

Regarding LS-SVM models, the optimization-value ranges for the regularization parameter γ and the RBF (radial basis function) kernel function parameter σ^2^ were set at 2^−1^–2^10^ and 2–2^15^, respectively, which were determined by applying a grid-search technique. For each combination of γ and σ^2^ parameters, the root mean square error of cross-validation (RMSECV) was calculated. The optimum parameters were selected when they produced the smallest RMSECV. Ninety samples in the prediction set were classified by the LS-SVM model with the optimal combinations of (γ, σ^2^).

For BPNN models, the optimal parameters of this matrix in modeling process were set as follows after the adjustments of parameters. The number of hidden layers, the dynamic parameter, the goal error and the times of training were set as 9, 0.6, 0.00001 and 1,000, respectively. The threshold error of recognition was also set as ±0.5.

For comparison, several LS-SVM and BPNN models were established using the selected PCs, KPCs and the textural variables as different inputs, respectively. Table 1 shows the discrimination results of six varieties of maize seeds in the calibration set and prediction set using these eight models, respectively. It could be seen that LS-SVM models generally performed better than BPNN models. Moreover, lower error rates were obtained in LS-SVM models when adding the textural features in discrimination models, while the prediction accuracy for BPNN models combined with textural features from GLCM was similar to that of the ones only using the spectral features (PCs and KPCs). Specifically, the wrong results of prediction set in PCA-LS-SVM and KPCA-LS-SVM models both happened in variety IV and V. A total of six samples were mistaken in each of these two models. However, only one and three samples were falsely judged by PCA-GLCM-LS-SVM and KPCA-GLCM-LS-SVM, respectively. Finally, the LS-SVM model combined with PCs by PCA and textural variables by GLCM obtained the best discrimination accuracy of 98.89% in this condition. Thus, the overall results indicated that the new combination of PCA-GLCM-LS-SVM exceeded the ones based on KPCA and BPNN in variety discrimination of maize seeds using VIS-NIR hyperspectral imaging. Probably because some maize seeds samples showing similar colour in different varieties would be wrongly discriminated if only using the spectral features to develop model. However, they can be identified more accurately using classification model based on the combination of spectral and textural features owing to the existence of some textural differences among them.

## 4. Conclusions

The above excellent discrimination results suggested that VIS-NIR hyperspectral imaging technique combined with PCA-GLCM feature extraction and LS-SVM could be successfully applied for conducting fast variety identification of commercial maize seeds. Three wavelengths (523, 579 and 863 nm) were selected as the optimum wavelengths according to first three PCs loading weights. Based on four textural features calculated from GLCM of each monochromatic image at optimal wavelengths, prediction accuracy of 98.89% was achieved using the LS-SVM calibration model, which was higher than that of using KPCA and BPNN calibration models. This increased accuracy is very important for discrimination of multiple varieties of maize seeds in mass and practical applications. Combining spectral features and texture features to establish LS-SVM discrimination models was proved as a prominent way for image classification with high accuracy. This finding will provide assistance for the future research of hyperspectral imaging analysis. Expanding the variety number and optimizing the image process algorithm should be put more effort in future study to validate the repeatability of the algorithms for real-time online applicability. Besides, more effective wavelengths would be acquired, which might be also important for the on-line inspection and portable instruments for commercial applications of adulteration detection.

## Figures and Tables

**Table 1.** Statistic result of discrimination models for prediction.

**Method**	**Accuracy of LS-SVM Model (%)**		**Accuracy of BPNN Model (%)**	
	**Calibration**	**Prediction**	**Calibration**	**Prediction**
PCA	95.00	93.33	94.58	91.11
PCA-GLCM	100	98.89	97.50	91.11
KPCA	93.75	93.33	93.33	91.11
KPCA-GLCM	99.58	96.67	98.33	90.00
